# Transcriptomics-driven identification of CDK1 as a central oncogenic driver in TNBC: an *in silico* structural modeling and MD simulation approach

**DOI:** 10.3389/fbinf.2026.1766384

**Published:** 2026-04-08

**Authors:** Uma Chaudhary, Sidharth Kumar Nanda Kumar, Magesh Ramaswamy, Mythili Asaithambi

**Affiliations:** 1 Department of Biotechnology, School of Biosciences and Technology, Vellore Institute of Technology (VIT), Vellore, Tamil Nadu, India; 2 Department of Biotechnology, Sri Ramachandra Institute of Higher Education and Research (DU), Chennai, Tamil Nadu, India; 3 Department of Sensor and Biomedical Technology, School of Electronics Engineering, Vellore Institute of Technology (VIT), Vellore, Tamil Nadu, India

**Keywords:** CDK1, cell cycle, molecular dynamics simulation, phytochemical, triple-negative breast cancer

## Abstract

**Introduction:**

Triple-negative breast cancer (TNBC) is an aggressive subtype that lacks ER, PR, and HER2 receptors, which limits the availability of targeted therapies. In this study, we analyzed CDK1 as a potential molecular target and evaluated natural compounds that might inhibit its activity.

**Methods:**

Transcriptomic comparison revealed 85 commonly upregulated mRNAs in TNBC, and functional enrichment combined with PPI network analysis indicated CDK1 as a major hub gene. To search for potential inhibitors, we screened an anticancer-focused phytochemical library from the SuperNatural 3.0 database using molecular docking followed by ADMET assessment.

**Results:**

Among the screened molecules, CID17584963 showed the strongest binding energy (−8.09 kcal/mol) and displayed pharmacokinetic properties comparable to or better than those of paclitaxel. Long-timescale (500 ns) molecular dynamics simulations further supported the stability of the CDK1–CID17584963 complex, with root mean square deviation (RMSD), root mean square fluctuation (RMSF), radius of gyration, solvent-accessible surface area (SASA), hydrogen-bond profiles, and principal component analysis (PCA) all indicating consistent interactions throughout the trajectory.

**Discussion:**

Taken together, these findings indicate that CID17584963 interacts with CDK1 more stably than the reference drug and may serve as a promising natural compound for further studies in TNBC therapy.

## 1 Introduction

Breast cancer (BC) is the most frequently diagnosed malignancy in women and continues to be a major cause of cancer-related mortality worldwide (Bray et al., 2024). Although therapeutic approaches have improved over the years, the substantial molecular diversity within BC continues to make effective management challenging. Based on the expression of three receptors, namely, HER2 (human epidermal growth factor receptor 2), ER (estrogen), and PR (progesterone), BC is categorized into luminal, HER2-enriched, and triple-negative subtypes (Turashvili and Brogi, 2017; Fukano et al., 2021). Among these, triple-negative breast cancer (TNBC) is particularly concerning. Defined by the absence of ER, PR, and HER2, TNBC accounts for approximately 10%–20% of BC cases and is associated with rapid disease progression, early metastasis, poor prognosis, and a significant lack of targeted therapeutic options (Garrido-Castro et al., 2019). Since TNBC does not express any hormone receptors and the currently available targeted therapies offer limited benefit, chemotherapy is the primary treatment. However, the frequent development of chemoresistance and the high rate of recurrence within 3–5 years after diagnosis are significant clinical challenges (Shimelis et al., 2018; Garrido-Castro et al., 2019). The biological heterogeneity within TNBC further complicates treatment decisions, emphasizing the need for reliable biomarkers and more personalized therapeutic approaches. Although prognostic and predictive markers could greatly aid precision medicine, the molecular drivers that define various TNBC subgroups are still not fully understood (Zhang et al., 2020).

Our transcriptomic analysis showed CDK1 to be a consistently upregulated gene in TNBC. CDK1 is a serine/threonine kinase and a core component of the cyclin-dependent kinase (CDK) family, well-known for its essential role in regulating cell-cycle progression (Qin and Li, 2025). Among the CDKs, CDK1 is chiefly important for the G2–M-phase transition, and its dysregulation has been linked to uncontrolled cell proliferation and tumor development (8). Aberrant CDK1 activity influences multiple oncogenic pathways, as shown by KEGG enrichment analyses, emphasizing its relevance as a core therapeutic target across various cancers (Izadi et al., 2020). KEGG enrichment analyses further support the involvement of CDK1 in multiple oncogenic signaling pathways, underscoring its relevance as a potential therapeutic target across different cancers (Wang et al., 2023). Previous studies also demonstrate that selective CDK1 inhibition, either alone or in combination with other anticancer therapies, can exert strong antitumor effects, including in breast cancer models (Izadi et al., 2020; Gupta et al., 2025). Despite the availability of conventional treatment procedures such as surgery, chemotherapy, and radiotherapy, resistance to therapy continues to cause breast cancer mortality. In addition, many modern anticancer agents cause considerable toxicity and impose economic and social burdens on patients; all these factors have renewed scientific interest in plant-derived compounds as alternative or complementary therapeutic options (Wali et al., 2025). Natural products have historically been used as medicinal agents, and accumulating evidence supports the anticancer properties of numerous phytochemicals. These compounds modulate key pathways such as MAPK and JAK/STAT3, offering safer and cost-effective alternatives to synthetic drugs (Wali et al., 2025). Natural polyphenols, in particular, have received attention for their capacity to influence oncogenic processes through antioxidant and anti-inflammatory mechanisms (Bhutta and Choi, 2025). Databases such as SuperNatural 3.0 now enable systematic exploration of bioactive phytochemicals, including the anticancer-focused libraries used in this study (Gallo et al., 2023).

In this work, we utilized a transcriptomics-driven computational workflow to identify CDK1 as a central oncogenic hub in TNBC. Using differential gene expression analysis, functional enrichment, and structure-based modeling, we analyzed the phytochemical candidates capable of targeting CDK1 through molecular docking, pharmacokinetic evaluation, and molecular dynamics simulation (MDS).

Furthermore, comparative assessments with the standard TNBC drug paclitaxel (taken as the control or reference) provide insights into potential drug-repurposing opportunities and support the development of natural compound-based therapeutic strategies for TNBC. Recent computational drug-discovery studies have utilized integrated *in silico* workflows incorporating ADMET prediction and molecular docking, thereby offering methodological precedence for the strategy used in this work (Alshehri et al., 2023; Alghamdi et al., 2024).

## 2 Methodology

### 2.1 Dataset retrieval and identification of differentially expressed mRNAs

We retrieved the dataset from the GEO database (Clough et al., 2024), and the keywords “Triple-Negative Breast Cancer,” “TNBC,” and “*Homo sapiens*” were used to search for the dataset. The criteria for the selection of the datasets of interest included (i) a higher number of TNBC samples and (ii) datasets with normal and control samples. Three microarray datasets that met these criteria are GSE61724, GSE45827, and GSE65194, comprising 68, 155, and 178 samples, respectively. The GEO2R tool was used to identify differentially expressed mRNAs (DE-mRNAs), where log_2_FC ≥ 2 and *p*-value ≤0.05 were considered significantly upregulated, whereas those with log_2_FC ≤ −2 and *p*-value ≤0.05 were considered significantly downregulated. These DE-mRNAs were visualized using a volcano plot (Davis and Meltzer, 2007). Subsequently, the GEPIA2 portal was utilized to extract the DE-mRNAs from the BRCA samples (based on the TCGA data), which were analyzed with a cutoff of log_2_FC (≥2 or ≤ −2) and *p*-value ≤0.05 (Tang et al., 2019). Common DE-mRNAs from the three microarray datasets and RNA-seq data from the TCGA–BRCA samples were identified using the InteractiVenn tool (Heberle et al., 2015). A total of 85 differential gene entries were extracted from those found at the intersection.

### 2.2 Enrichment analysis and PPI network

Pathway enrichment analysis was performed using SRplot (Tang et al., 2023), an online visualization platform that supports Gene Ontology (GO)- and KEGG-based enrichment analysis. DE-mRNAs were input to identify statistically enriched pathways, which were visualized through bubble and network plots. To reveal key functional associations and biological mechanisms, network relationships among pathways were displayed based on shared gene components. This tool contains many functions, such as transcriptomics, which includes the GO pathway enrichment analysis module. This module combined clusterProfiler and pathview. The most widely used STRING tool was used to construct protein–protein interaction (PPI) networks, which help analyze the relationships between significantly upregulated and downregulated common DE-mRNAs. Here, we first selected multiple proteins, pasted a list of gene names or symbols, or uploaded a file, selected the organism, searched for PPIs, and continued building the network. The high confidence level for the network is set to 0.7 (Szklarczyk et al., 2023).

### 2.3 Retrieval and preparation of the CDK1 structure

The 3D structure of the CDK1 gene in PDB format was obtained from the Protein Data Bank (PDB) database (Berman, 2000). The selected PDB ID: 5LQF has a resolution of 2.06 Å and a sequence length of 302 amino acids. Protein preparation steps include removal of all 1. heteroatoms, 2. extra side chains, and 3. water molecules from the target complex using AutoDock, followed by energy minimization in a vacuum after adding polar hydrogens using the AutoDock tool (Discovery Studio, 2015). The optimized PDB models were saved in the pdbqt format. Additionally, the involvement of CDK1 in immune responses was examined using TISIDB, and its expression levels were analyzed across normal, tumor, and metastatic stages using TNMplot.

#### 2.3.1 Active site prediction in protein

The catalytic residues involved in ligand binding on the target protein surfaces were identified using CASTp ver. 3.0, an online binding-site prediction tool; then, virtual screening and molecular docking were performed with the ligand library (Tian et al., 2018). CASTp quantitatively measures the area and volume of each pocket present on the protein surface. The target protein structure in PDB format was uploaded to the server or provided using a PDB ID. While maintaining the default probe sphere radius of 1.4 Å around the protein, the predicted active site amino acid residues, along with their sequence IDs, were displayed in the tabular data and recorded for subsequent docking analyses.

### 2.4 Ligand retrieval and preparation

In this study, we retrieved anticancer natural products (ANPs) from SuperNatural 3.0, a freely accessible and reliable database of natural products and their derivatives. This database provides detailed information on the physicochemical properties, toxicity class, mechanism of action (MoA), therapeutic pathways, disease indications, focused-targeted libraries, and taste-related attributes (Gallo et al., 2023). The use of ANPs aids in identifying new leads for drug discovery, and a total of 100 ANPs were extracted from the anticancer library of the SuperNatural 3.0 database. These 3D-SDF structures were then organized, and energy-minimization was performed with MMFF94 force field for 1,000 steps using the Open Babel tool (O’Boyle et al., 2011).

### 2.5 Virtual screening and ADME and toxicity prediction

Computer-aided drug design (CADD) is a commonly employed virtual molecular screening method used for identifying lead compounds with desirable biological activity. Virtual screening involves finding the strongest binding energy molecules by docking libraries of small molecules to the target macromolecules. This study utilized the freely available version of PyRx software to screen the natural compounds against CDK1. Compounds showing the least binding energies compared to the control drug were shortlisted for further evaluation (Dallakyan and Olson, 2015). The selected phytocompounds were profiled based on the ADMET characteristics to assess their drug-like properties. SwissADME was used to evaluate the absorption, distribution, metabolism, and excretion (ADME) parameters (Daina et al., 2017), while toxicity prediction was carried out using the ProTox-III online tool. In our screening, the selection of the lead compounds for docking and MDS was not based solely on the binding affinity. Further analysis was performed based on consideration of only compounds that met the ProTox toxicity criteria (class 5 or above) (Banerjee et al., 2024).

### 2.6 Molecular docking

The preparation of selected ligand–protein complexes was carried out using AutoDockTools version 4.2.6. In this tool, the protein structures’ PDB file was first imported, followed by the generation of the grid box to include all amino acid residues within the predicted binding pockets, as identified using CASTp. Parameters for localization, spacing, and dimensions were carefully defined (Tian et al., 2018), as previously described. A ligand library of 100 anti-cancer compounds was subjected to virtual screening using PyRx, followed by targeted docking with AutoDock Vina (Morris et al., 2009). For the comparison analysis against the anti-cancer compounds, paclitaxel was included as a control within the library. Finally, the selected compounds were visualized for 2D interactions using the BIOVIA Discovery Studio Visualizer (Discovery Studio, 2015). The interacting residues between the ligand and protein confer the optimal docked poses for further examination. Out of the selected list of top ligands, we chose a compound that exhibited the highest negative-binding energy compared to the control drug for subsequent MDS analysis.

### 2.7 Molecular dynamics and post MD analysis

MDS was performed on the best docking poses to evaluate the stability of the protein–ligand interactions. Input files for MDS were generated using SwissParam, which applied the CHARMM force field parameters for proteins. Ligand topology files were prepared via SwissParam using the CHARMM General Force Field. The first step involved reading the coordinates of the protein–ligand complex, which was then solvated in a cubic box containing TIP3P water molecules. The second step includes solvation of the complex, where the system’s shape and size are defined, and Na^+^ and Cl^−^ ions are added to neutralize the charge by replacing water molecules. In the third step, periodic boundary conditions (PBCs) were applied to the whole system’s geometry. Non-covalent interactions were considered with a 12 Å cutoff, neighbor lists were updated using the Verlet cutoff scheme, and extended electrostatics were computed using the particle mesh Ewald (PME) method. The CHARMM27 force field was applied to all protein–ligand complexes. Before production runs, systems underwent energy optimization using the steepest descent algorithm for 5,000 steps. The fourth and fifth steps involve equilibration and production, which were conducted under NVT and NPT ensembles. To achieve this, the system was equilibrated at 300.15 K for 50,000 ps and pressure was applied using positional restraints of 400 kJ/mol·nm^2^ and 40 kJ/mol·nm^2^ on the backbone and side chains, respectively. In the final step, production MDSs were carried out for 500 ns in the NPT ensemble at 300.15 K and 1 bar using the Nose–Hoover thermostat for temperature control and the Parrinello–Rahman barostat for pressure regulation. The LINCS algorithm was used to constrain hydrogen bonds, and the V-rescale thermostat (300 K, 1 ps coupling constant) was employed to maintain system stability. Trajectories were stored every 2 ps throughout the 500 ns production run (El Rhabori et al., 2024). All MDS, including equilibration and production runs, were executed using GROMACS version 2020.2 (Páll et al., 2020).

### 2.8 PCA plot

In this work, principal component analysis (PCA) was applied to map the structural landscape of the protein when it binds to paclitaxel (control) and to the test screened compounds. The analysis allowed us to examine how the overall atomic motions vary across the complexes. In the PCA plot, the eigenvectors describe the direction of the dominant motions, while the eigenvalues correspond to the extent of these fluctuations. The principal components for each complex were obtained by diagonalizing the covariance matrix. Typically, the first few components capture most of the large-scale movements of the protein–ligand system (Amadei et al., 1993). For this study, GROMACS was used to build the PCA plot and diagonalize the covariance matrix from “gmx.covar,” and eigenvectors were extracted from “gmx.anaeig.” The trajectories were finally projected. All 2D PCA plots were generated using the XMGrace tool.

### 2.9 FEL plot

Free energy landscape (FEL) analysis was performed using PCA based on the covariance matrix of atomic fluctuations obtained from the MD trajectory. The first two principal components (PC1 and PC2) were used as reaction coordinates, and Gibbs free energy was calculated using the Boltzmann distribution formula: ΔG = −kBT ln P, where kB is the Boltzmann constant, T is the temperature, and P is the probability distribution (Homeyer and Gohlke, 2012).

### 2.10 Free energy perturbations

The free energy profile was computed from the molecular dynamics trajectory using the Boltzmann distribution according to the equation ΔG = −kBT ln P, where kB is the Boltzmann constant, T is the simulation temperature, and P represents the probability distribution along the chosen reaction coordinate. The analysis was performed using MD trajectory post-processing tools (Homeyer and Gohlke, 2012).

### 2.11 DCCM analysis

Dynamic cross-correlation matrix (DCCM) analysis was performed to examine correlated motions between protein residues during the MD simulation using GROMACS (gmx.covar). The cross-correlation coefficients were calculated based on the displacement of Cα atoms using the equation: Cij = ⟨Δri · Δrj⟩/(⟨Δri^2^⟩⟨Δrj^2^⟩)^1/2^, where Δri and Δrj represent the positional fluctuations of residues i and j, respectively (dos Santos Nascimento et al., 2022).

## 3 Results

### 3.1 Identification of mRNAs with differential gene expression

**FIGURE 1:**
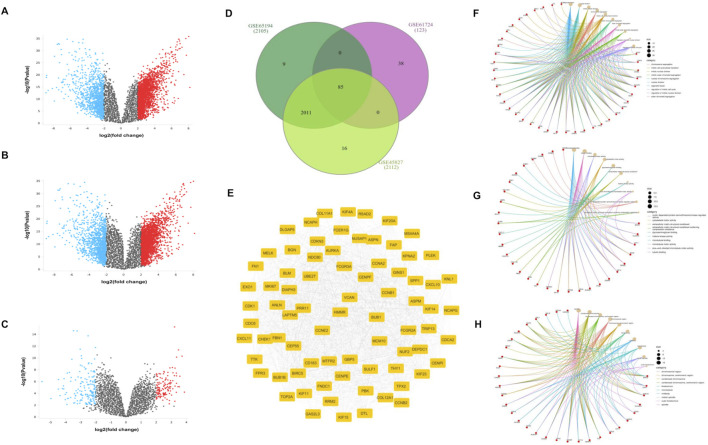
Volcano plots of GSE61725, **(A)** GSE45827, **(B)** GSE65194, and **(C)** GSE61724; **(D)** a total of 85 common DEGs shown in the intersection of the Venn diagram.; **(F)** biological processes, **(G)** molecular functions, and **(H)** cellular components.

In this study, the GSE61724, GSE45827, and GSE65194 datasets were obtained from GEO and analyzed through the GEO2R platform to identify DE-mRNAs in TNBC. Genes with a *p*-value ≤0.05 and an absolute log_2_ fold change of ≥2 were considered significantly upregulated, whereas those with log_2_ fold changes ≤ −2 were treated as significantly downregulated. The DEGs obtained from each dataset were visualized using volcano plots, as shown in Figures 1A–C. To complement this, differential expression data from the TCGA–BRCA cohort were extracted using GEPIA2, which produced 3,554 gene entries. A Venn intersection formed using InteractiVenn showed 85 commonly upregulated DEGs that were shared across the three GEO datasets and the TCGA–BRCA dataset, as illustrated in Figure 1D.

### 3.2 Functional enrichment analysis and PPI network

**FIGURE 2:**
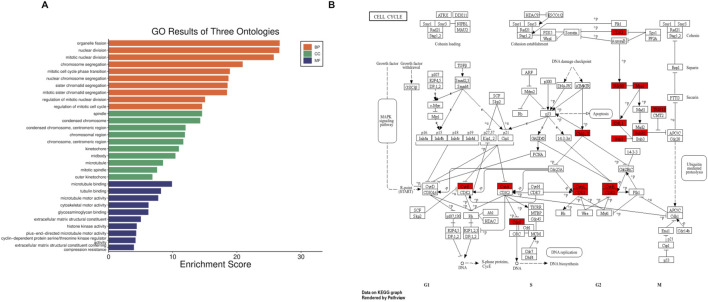
**(A)** Gene ontologies for DEGs: biological processes (BPs), molecular functions (MFs), and cellular components (CCs); **(B)** KEGG pathway, where the genes are highlighted in (red) the cell-cycle pathway.

GO analysis was carried out for the 85 DE-mRNAs to identify the major biological processes and functional categories associated with TNBC. The SRplot web server was used to generate the GO enrichment results, including biological process (BP), molecular function (MF), and cellular component (CC) Circos plots, which are shown in Figure 2A. KEGG pathway enrichment was also performed, and the significant pathways (p < 0.05) are displayed in Figure 2B. In addition, a PPI network for the 85 genes was constructed using the STRING database, and the resulting network is presented in Figure 1E.

### 3.3 Protein structure retrieval and preparation

**FIGURE 3:**
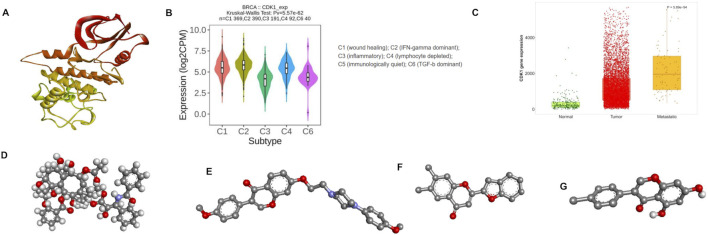
**(A)** 3D-structure of CDK1; **(B)** TISIDB—indicating the expression of CDK1 in immune subtypes in BRCA; **(C)** TNM plot showing CDK1 expression in all three stages; **(D)** three-dimensional structure of paclitaxel; **(E)** three-dimensional structure of CID17584963; **(F)** three-dimensional structure of CID740749; **(G)** three-dimensional structure of CID5398360.

The 3D structure of the CDK1 protein used in this study, shown in Figure 3A, was selected after reviewing the DEGs results and relevant literature. The structure was then downloaded in the PDB format. Active-site prediction and protein preparation were carried out using the CASTp server, which enabled the identification of the key residues forming the binding pocket. CDK1’s possible involvement in immune-related processes is illustrated in Figure 3B, and its expression profile across normal, tumor, and metastatic samples is displayed in Figure 3C.

### 3.4 Ligands retrieval, virtual screening, and ADMET prediction

Virtual screening was carried out using PyRx. The ligand library consisted of 100 phytochemicals collected from the SuperNatural 3.0 database. All compounds were examined in SwissADME to assess the basic pharmacokinetic features such as Lipinski compliance, gastrointestinal absorption, BBB permeability, and potential interaction with P-glycoprotein. Toxicity predictions, including carcinogenicity, cytotoxicity, immunotoxicity, and mutagenicity, were obtained from the ProTox-II tool, and the results are summarized in Table 1. Based on the ADMET evaluation, three phytochemicals satisfied the selection criteria. These compounds were chosen because they showed good predicted properties. These shortlisted compounds in Figures 3E–G were exported in the 3D-SDF format and converted into PDBQT files using the Open Babel GUI for docking with AutoDock Vina.

**TABLE 1:** Virtual screening and ADMET results of the phytocompounds.

S. No	Ligand	SMILES	SwissADME	PROTOX
1	3-(4-methoxyphenyl)-7-(2-(4-(4-methoxyphenyl)piperazin-1-yl)ethoxy)-4H-chromen-4-one	COC1 = CC = C(C=C1)C2 = COC3 = C(C2 = O)C=CC(=C3)OCCN4CCN(CC4)C5 = CC = C(C=C5)OC	No violations	5
2	2-(1-Benzofuran-2-yl)-6,7-dimethyl-4H-chromen-4-one	CC1 = CC2 = C(C=C1C)OC(=CC2 = O)C3 = CC4 = CC = CC = C4O3	No violations	5
3	3-(4-Chlorophenyl)-5,7-dihydroxy-4H-chromen-4-one	C1 = CC(=CC = C1C2 = COC3 = CC(=CC(=C3C2 = O)O)O)Cl	No violations	5

### 3.5 Molecular docking

Protein–ligand docking helps identify whether a compound can form meaningful interactions with a target protein. In this work, lower binding energies were taken as an indication of stronger affinity toward CDK1. Protein preparation and grid generation followed standard procedures, and the docking protocol was validated by redocking a ligand into the known binding pocket of CDK1 (PDB ID: 5LQF). The grid parameters used for docking were applied in AutoDock Vina to screen a set of 100 phytochemicals along with the reference drug paclitaxel. All docking runs were carried out in triplicate, and the average scores for each compound were calculated. The docked complexes were examined in BIOVIA Discovery Studio. As summarized in Table 1, the screened phytochemicals exhibited better binding energies than paclitaxel (3D structure shown in Figure 3D). Among them, CID17584963, CID740749, and CID5398360 exhibited consistently higher affinities across the evaluated receptors.

**FIGURE 4:**
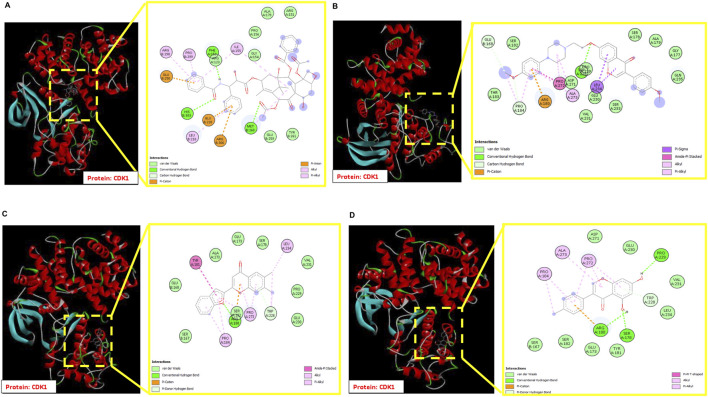
Three-dimensional molecular interaction of CDK1 and ligands, enlarged schematical two-dimensional representation of interacting residues of CDK1 with **(A)** paclitaxel, **(B)** CID17584963, **(C)** CID740749, and **(D)** CID5398360.

Binding energy is an important indicator of the stability of the interactions within a protein–ligand complex, with more negative values indicating stronger binding and greater therapeutic potential. The three selected phytochemicals recorded binding energies of −8.09, −7.69, and −6.70 kcal/mol, while paclitaxel showed a binding energy of −5.59 kcal/mol against CDK1. Visualization of the CDK1–CID17584963 complex showed several stabilizing contacts. These included one conventional hydrogen bond with TRP228, carbon–hydrogen bonds with GLU168 and PRO184, and a range of van der Waals interactions involving THR183, SER182, VAL231, ASP271, GLU230, PRO229, SER178, ALA179, GLY177, and GLN235. Additional interactions such as amide–π stacking with PRO272, π–cation interaction with ARG180, π–sigma interaction with LEU234, alkyl interaction with PRO184, and π–alkyl interaction with ALA273 were also observed. In contrast, the CDK1–paclitaxel complex formed three hydrogen bonds with HIS303, MET260, and PHE153. The broader set of interactions formed by CID17584963 likely contributed to its higher binding energy. Details of these interaction profiles are listed in Table 2. Two-dimensional interaction diagrams are shown in Figure 4. Considering the overall docking results, CID17584963 showed the most favorable interaction pattern. Therefore, it was chosen for further investigation through MDS.

**TABLE 2:** Molecular interactions of CDK1 with paclitaxel, CID17584963, CID740749, and CID5398360.

S.No	Protein–ligand complex	Docking score (kcal/mol)	Conventional H-bond and carbon–hydrogen bond	Other interactions
1	CDK1–paclitaxel	−5.59	HIS303, MET260, PHE153, and ARG123	VD: GLY154, PRO156, ALA179, ARG151, GLU259, and TYR261; Pi–anion: GLU258, GLU220, and ARG306; Pi–alkyl: ARG298, PRO299, ILE155, and MET260
2	CDK1–CID17584963 [3-(4-methoxyphenyl)-7-(2-(4-(4-methoxyphenyl)piperazin-1-yl)ethoxy)-4H-chromen-4-one]	−8.09	TRP228, GLU168, and PRO184	VD: THR183, SER182, VAL231, ASP271, GLU230, PRO229, SER178, ALA179, GLY177, and GLN235; Pi–cation: ARG180; amide–Pi-stacked: PRO272; Alkyl: PRO184; Pi–alkyl: ALA273, Pi–sigma: LEU234
3	CDK1–CID740749 [2-(1-benzofuran-2-yl)-6,7-dimethyl-4H-chromen-4-one]	−7.69	ARG180 and TRP228	VD: SER167, GLU168, ALA273, SER182, TRP228, GLU173, SER178, GLU230PRO229, and VAL231; Pi-cation: SER182; amide–Pi-stacked: TYR181; alkyl: TRP228 and LEU234, Pi–alkyl: PRO184 and PRO272
4	CDK1–CID5398360 [3-(4-chlorophenyl)-5,7-dihydroxy-4H-chromen-4-one]	−6.7	ARG180, SER178, and PRO229	VD: SER167, SER182, GLU173, TYR181, LEU234, VAL231, GLU230, and ASP271; Pi–cation: ARG180; Alkyl: PRO184; Pi–alkyl: PRO184, ALA273, PRO272

### 3.6 Molecular dynamics of the CDK1 protein

**FIGURE 5:**
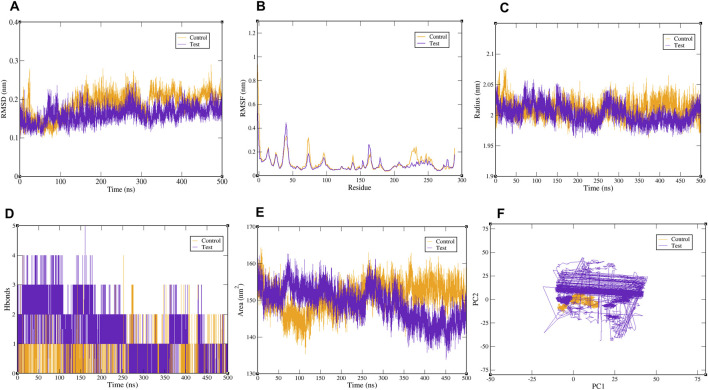
MDS trajectories generated over a 500 ns simulation period, where CDK1 is complexed with paclitaxel (control) and CID17584963 (test) ligands. **(A)** RMSD plot, **(B)** RMSF plot, **(C)** Rg plot, **(D)** number of H-bond plot, **(E)** SASA plot, and **(F)** PCA plot.

In this work, a 500 ns MDS was performed for the CDK1–paclitaxel (control) and CDK1–CID17584963 (test) complexes using GROMACS 2020.2, as shown in Figure 5. RMSD was used to track the overall structural deviation from the starting conformation during the simulation. Lower RMSD values generally reflect a more stable complex, while higher values indicate greater movement. Both systems reached a steady state at approximately 50 ns and remained mostly stable until approximately 400 ns, as shown in Figure 5A. The CDK1–CID17584963 complex showed minor deviations near 150 ns and 380 ns, with small fluctuations toward the end of the trajectory. The CDK1–paclitaxel complex showed a fluctuation pattern with noticeable movements at approximately 150, 330, 380, and 410 ns. Throughout the trajectory, the RMSD values for both the test and control complexes remained within the range of 0.1 nm–0.2 nm, indicating acceptable stability for the complex. To examine the residue-level flexibility of CDK1 during ligand binding, RMSF was calculated. Lower RMSF values indicate reduced residue mobility and a more stable complex. Residues spanning positions 0–302 were evaluated for both systems. As shown in Figure 5B, both ligand-bound complexes followed nearly identical fluctuation trends. This suggests that neither CID17584963 nor paclitaxel induced significant local perturbations in the protein backbone, and both maintained comparable residue stability during binding. To assess the protein compactness, the radius of gyration (Rg) was analyzed. More compact proteins generally showed lower radius of gyration values and improved structural stability on the backbone. Across the simulation, Rg values for the two complexes remained between 1.95 and 2.10 nm, as shown in Figure 5C. Although both systems displayed stable compactness, the CDK1–CID17584963 complex consistently showed slightly lower Rg values, indicating marginally stronger packing of the protein around this ligand. To evaluate the stability of ligand binding to the protein, hydrogen-bond analysis was also carried out. The CDK1–CID17584963 complex maintained 2–4 hydrogen bonds throughout the simulation, whereas the CDK1–paclitaxel complex maintained 1–3, as shown in Figure 5D. The higher number of hydrogen bonds in the CID17584963 complex indicates stronger interactions and enhanced stability within the complex. To examine solvent exposure during the simulation, solvent-accessible surface area (SASA) analysis was carried out. Both complexes displayed SASA values in the range of 140 nm^2^–160 nm^2^ over 500 ns, as illustrated in Figure 5E. The CDK1–CID17584963 complex showed slightly lower SASA values, suggesting a more compact and less solvent-exposed conformation than the CDK1–paclitaxel complex. PCA was performed to evaluate the global motions of the protein–ligand systems. The PCA plots for both complexes are shown in Figure 5F. A more restricted conformational space reflects reduced mobility and greater structural stability. The CDK1–paclitaxel complex showed a slightly narrower distribution, while the CDK1–CID17584963 complex showed a similar but slightly broader range of motion without any major deviations. Overall analysis showed that both ligands effectively stabilized CDK1, with CID17584963 exhibiting greater stability than the control drug.

When combined, all the analyses, including RMSD, RMSF, Rg, SASA, hydrogen-bond profiles, and PCA, indicate that both ligands formed stable interactions with CDK1 throughout the simulation. However, the CDK1–CID17584963 complex consistently displayed slightly better stability and compactness than the CDK1–paclitaxel complex. These overall observations indicate that CID17584963 has a strong binding profile and may represent a promising candidate for CDK1-targeted therapeutics in TNBC.

### 3.7 Free energy analyses and dynamic cross-correlation findings

**FIGURE 6:**
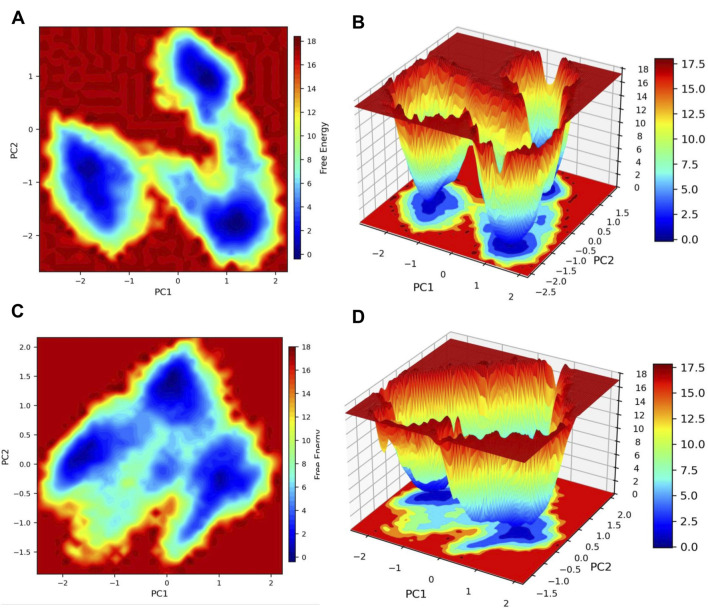
Two-dimensional free energy landscapes of the system projected onto PC1 and PC2, shown as contour maps **(A)** and corresponding 3D surfaces **(B)** for the control compound and the contour maps **(C)** and corresponding 3D surfaces **(D)** for the test compound. Low-energy basins (blue) represent stable conformations, while high-energy regions (red) indicate barriers separating these states.

**FIGURE 7:**
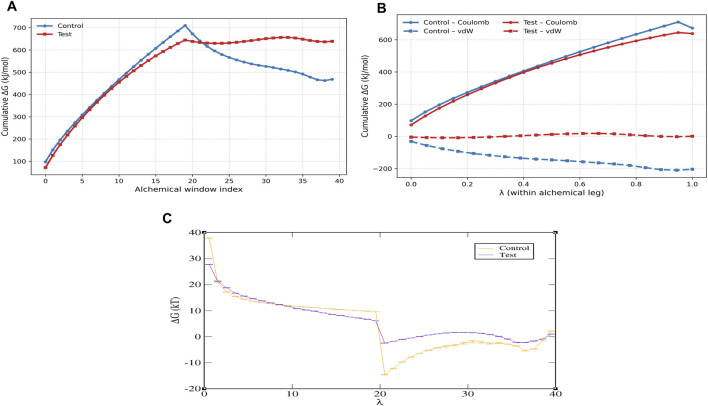
Free energy perturbation of the protein–ligand complex from molecular dynamics simulations. **(A)** Cumulative FEP, **(B)** control vs. test: Coulomb and van der Waals FEP, and **(C)** free energy perturbation control vs. test. The profile shows a pronounced global minimum, indicating a thermodynamically stable conformational state, while higher-energy regions correspond to less favorable conformations.

**FIGURE 8:**
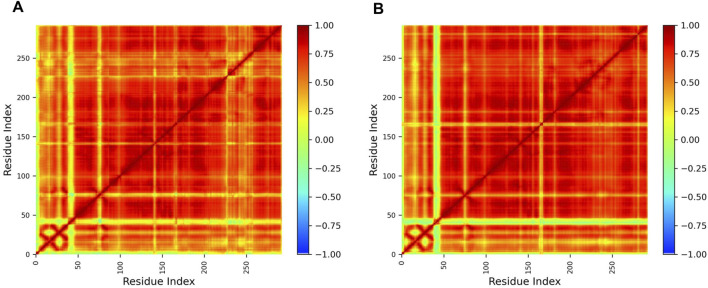
Dynamic cross-correlation matrix of the protein–ligand complex from molecular dynamics simulations. **(A)** Control DCCM and **(B)** test DCCM. The red and blue regions indicate the positively and negatively correlated Cα residue motions, respectively, revealing coordinated movements and ligand-induced stabilization.

The free energy landscape analysis (Figure 6) revealed a well-defined global minimum, indicating that the protein–ligand complex predominantly occupies a stable conformational state during the simulation. Consistently, the free energy perturbations (Figure 7) exhibited a pronounced low-energy minimum, further supporting the thermodynamic stability of the complex throughout the trajectory. In addition, DCCM analysis (Figure 8) showed strong positive correlations among key residue clusters, indicating coordinated internal motions within the protein structure upon ligand binding.

## 4 Discussion

This study integrates gene expression data, enrichment analysis, virtual screening, docking, and long-timescale MDS to understand how CDK1 is regulated in TNBC and find a natural compound that may bind effectively with the target. From the selected GEO and the TCGA–BRCA cohort datasets, we identified 85 commonly upregulated genes, with CDK1 consistently appearing among the key genes associated with cell-cycle activity in GO and pathways. This corroborates earlier findings showing that CDK1 is upregulated in TNBC and contributes to rapid tumor progression.

The GO and KEGG enrichment results also highlighted pathways such as cell-cycle control, DNA repair, and mitotic progression, which further indicate CDK1’s involvement in TNBC biology. The PPI network analysis showed that CDK1 interacts with several other key regulatory genes, reinforcing its central role in this cancer type. Because of this, CDK1 was selected for structural analysis and ligand-binding studies. CDK1 is a major regulator of the G2/M checkpoint, and TNBC cells often depend heavily on mitotic kinases for their rapid growth. This kinase exhibited immune-related activity responses and showed variable expression at different cellular stages, such as during cancer. High CDK1 expression has also been associated with poor prognosis and reduced response to chemotherapy in some patients. Blocking CDK1 activity may, therefore, slow down mitotic entry and may even help overcome resistance seen with microtubule-targeting drugs such as paclitaxel (Izadi et al., 2020; Sofianidi et al., 2024).

Virtual screening of ANPs led to the identification of three ANPs that passed the ADMET filters. Among them, CID17584963 showed the best docking score against CDK1 when compared with paclitaxel. The interaction analysis also showed that CID17584963 formed several types of contacts, including hydrogen bonds, van der Waals forces, and π-based interactions, which may be responsible for its stronger binding energy. In contrast, paclitaxel formed fewer interactions, which may explain its slightly weaker docking performance in this context.

Earlier computational and experimental studies have explored various CDK1 inhibitors, including small synthetic molecules and ATP-competitive analogs (Sofianidi et al., 2024; Teotia et al., 2024). Some natural compounds have also been proposed as CDK1 modulators, although such reports remain limited. Compared with these previous findings, CID17584963 showed comparable or better predicted affinity in our study. Paclitaxel, which is currently used in TNBC treatment, does not directly target CDK1 but influences mitosis through microtubule stabilization (Nakayama et al., 2009). Our results suggest that CID17584963 may act through a different mechanism by interacting with CDK1. These findings align with earlier work emphasizing the therapeutic relevance of targeting mitotic regulators in TNBC (Izadi et al., 2020).

The molecular dynamics simulations provided additional support for the binding of CID17584963 to CDK1. The RMSD plots showed that both CDK1–CID17584963 and CDK1–paclitaxel remained stable for most of the 500 ns simulation period. RMSF values also indicated similar residue-level stability for both systems. The Rg and SASA results showed that CID17584963 maintained a slightly more compact and less solvent-exposed protein structure. The hydrogen-bond analysis also favored CID17584963 as it maintained approximately 2–4 hydrogen bonds throughout the simulation, while paclitaxel maintained a slightly lower number of bonds. PCA plots indicated that both ligands maintained the protein within a restricted conformational space, with minimal large-scale motion. In summary, these observations suggest that CID17584963 forms a stable bond with CDK1 and preserves structural compactness throughout the simulation period.

The free energy analyses collectively indicate that the protein–ligand complex attains a thermodynamically stable conformational state during the simulation. The presence of a single dominant low-energy basin in the FEL, together with a pronounced global minimum in the free energy perturbations, indicates restricted conformational transitions and favorable binding stability. While PCA captures the dominant collective motions sampled by the system, the FEL identifies the energetically preferred conformations, highlighting their complementary roles in describing dynamic and thermodynamic behavior. Furthermore, DCCM analysis revealed enhanced positively correlated motions and reduced anti-correlated movements upon ligand binding, indicating coordinated residue dynamics and the overall structural stabilization of functionally important regions.

The stability of the CDK1–CID17584963 complex observed during the MD simulation can be described by the 2D-interaction pattern among the residues identified in docking. The compound CID17584963 might occupy the ATP-binding pocket and form contacts with several key residues, such as TRP228, GLU168, and PRO184. The presence of non-covalent interactions, such as π-interactions and multiple van der Waals contacts, is a likely factor contributing to its stronger packing around the ligand. Such interactions might stabilize the hinge region and the active-site loop of CDK1, thus reducing local fluctuations during the simulation. In contrast, paclitaxel formed fewer stabilizing contacts, which may explain its slightly lower stability in certain MDS parameters.

Overall, the combined results from docking and MDS indicate that CID17584963 has a better binding stability than paclitaxel toward CDK1. Since CDK1 is already known to be overexpressed and highly active in TNBC, a compound that can bind to it strongly may serve as a good starting point for developing new therapeutic approaches. Based on the computational analysis, CID17584963 appears to be a promising candidate for future experiments.

Although the results are encouraging, this is an entirely computational study, which has its limitations. The actual biological activity of CID17584963 is not yet known, and both *in vitro* and *in vivo* experimental validation are required. CDK1 is an essential cell-cycle serine/threonine kinase, which raises concerns about potential toxicity in normal dividing cells (Wang et al., 2023). The ADMET predictions also may not fully match real-life absorption or metabolism. Moreover, docking and MD simulations do not address selectivity toward CDK1 compared with other CDKs, which should be tested further in wet-lab experiments. Future work should include *in vitro* CDK1 inhibition assays and cytotoxicity studies on TNBC cell lines to verify the compound’s activity. It will also be useful to check off-target effects on other CDKs (Sofianidi et al., 2024). Structural optimization of CID17584963 may further improve its potency and selectivity. Finally, validating the compound in animal models could help determine its real therapeutic effects.

In this work, an integrated transcriptomic and computational framework was applied to identify CDK1 as a key oncogenic regulator in triple-negative breast cancer. With this approach, we identified an ANP named CID17584963 as a promising therapeutic candidate. The molecule exhibited characteristics such as strong binding energy, robust structural stability, and favorable pharmacokinetics when targeted against CDK1. Both molecular docking and extended molecular dynamics simulations confirmed that this ANP establishes stable and specific interactions within the CDK1 pocket, maintaining structural integrity compared with the control drug paclitaxel.

Overall, observations indicate that CID17584963 has the potential to modulate CDK1-dependent pathways implicated in cell-cycle progression, DNA repair, and apoptotic regulation. This makes it a possible natural alternative or complementary agent to existing TNBC treatments. A limitation of this study is the absence of re-docking or selectivity validation against additional CDK family members, which may be addressed in future studies to further refine the binding specificity.

However, as the current findings are derived solely from *in silico* analyses, comprehensive *in vitro* (e.g., qRT-PCR, Western blot, and functional assays in TNBC cell lines) and *in vivo* studies are required to validate their biological activity, therapeutic safety, and clinical applicability. Overall, our study provides a foundation for future experimental investigation of phytochemical-based interventions targeting CDK1 in TNBC.

## Data Availability

The datasets presented in this study can be found in online repositories. The names of the repository/repositories and accession number(s) can be found in the article/supplementary material.
